# Psychological intimate partner violence against women in the European Union: a cross-national invariance study

**DOI:** 10.1186/s12889-019-7998-0

**Published:** 2019-12-27

**Authors:** Manuel Martín-Fernández, Enrique Gracia, Marisol Lila

**Affiliations:** Department of Social Psychology, University of Valencia, Avda. Blasco Ibañez 21, 46010 Valencia, Spain

**Keywords:** Intimate partner violence, Psychological violence, Emotional abuse, Controlling behavior, Measurement invariance, Cross-national research, European Union, Surveys

## Abstract

**Background:**

Intimate partner violence against women (IPVAW) is a worldwide public health problem. One of the most frequent forms of this type of violence in western societies is psychological IPVAW. According to the European Union (EU) Fundamental Rights Association (FRA) the prevalence of psychological IPVAW in the EU is 43%. However, the measurement invariance of the measure addressing psychological IPVAW in this survey has not yet been assessed.

**Methods:**

The aim of this study is to ensure the cross-national comparability of this measure, by evaluating its measurement invariance across the 28 EU countries in a sample of 37,724 women, and to examine how the levels of this type of violence are distributed across the EU.

**Results:**

Our results showed that the psychological IPVAW measure presented adequate psychometric properties (reliability and validity) in all countries. A latent structure of one factor was supported and scalar invariance was established in all countries. The average levels of psychological IPVAW were higher in countries like Finland, Latvia, Lithuania, and Sweden compared to the rest of the EU countries. In many of the other countries the levels of this type of violence overlapped.

**Conclusion:**

Our findings underlined the importance of using appropriate statistical methods to make valid cross-national comparisons in large population surveys.

## Background

Intimate partner violence against women (IPVAW) is a worldwide social and public health problem [1–3], with serious consequences not only for the victims’ physical and psychological well-being, but also for their children, and the wider community [4–7].

One of the most frequent forms of this type of violence in western societies is psychological IPVAW, which can occur either in isolation, or in conjunction with other forms of intimate partner violence [8–11]. There is, however, a strong link between psychological and physical IPVAW, since psychological violence often precedes physical IPVAW, and it is considered one of its main risk factors [12, 13].

The average prevalence of psychological IPVAW in large population surveys varies largely depending on how this type of violence is defined and measured, with some studies estimating its prevalence at around 10–20% while others found prevalence rates of around 80–90% [14–20]. The frequency and severity of psychological IPVAW can also differ widely from one country to another [2, 14], implying that the way in which this type of violence is perceived and interpreted can vary across countries and cultures.

Most surveys addressing psychological IPVAW have followed the tradition of the Conflict Tactics Scale (CTS) in defining it through specific behaviors [2, 21, 22]. Two aspects are usually considered in the assessment of this type of violence: emotional abuse and controlling behavior [11, 12, 23–25]. Emotional abuse involves behaviors intended to generate emotional harm or threat of harm, such as belittling, humiliating, threatening or intimidating the victim, whereas controlling behavior entails monitoring partner’s behaviors or isolating them by limiting actions, such as forbidding them to leave the house, restricting contact with other people, or continually insisting on knowing the victim’s whereabouts [2, 14, 26, 27].

In the European Union (EU), a survey conducted by the EU Agency for Fundamental Rights (FRA) across the 28 member countries found that the average prevalence of psychological IPVAW was 43%, ranging from 31% in Ireland to 60% in Latvia [26]. The main advantage of this survey is that it also followed the CTS tradition, using the same set of questions addressing emotional abuse and controlling behavior in the 28 EU member states. Nevertheless, as is the case in most surveys, the measurement invariance of these questions has not yet been assessed, which calls into question the validity of these cross-national comparisons. It is therefore not possible to ensure whether the differences in psychological IPVAW prevalence across the EU countries reflects actual differences between countries, or whether they are the result of different cultural beliefs or expectations about intimate partner violence that may distort the interpretation of the FRA survey questions.

Therefore, before making any comparison across countries it is necessary to address the measurement invariance of the set of questions used in different countries [28–30]. Measurement invariance is an important prerequisite in cross-national research as it allows meaningful comparisons to be made across countries by ruling out the possibility of cultural bias in the respondents’ answers [31–33]. When measurement invariance is not supported, it cannot be assumed that respondents from different countries interpret and answer the questions in the same way, and hence their scores cannot be directly compared [34]. Thus, obtaining prevalence rates to compare samples from different countries without first assessing measurement invariance could lead to inaccurate and biased conclusions, since the validity of such comparisons may become compromised.

The principal aim of this study is to ensure the cross-national comparability of the set of questions addressing psychological IPVAW used in the FRA survey, by evaluating whether respondents of each country conceptualize and interpret these questions in the same way. For validity purposes, we also examined the relationships of psychological IPVAW to other forms of partner violence, such as physical and sexual IPVAW, and to other related sociodemographic and background variables, such as self-perceived health, household income, and experiences of child abuse [4, 11, 13, 35–39]. Once the measurement invariance of this measure and his validity were established, we aim to make valid and appropriate comparisons of the psychological IPVAW levels across all EU countries.

## Methods

### Participants

The sample used in the present study consisted of the responses of 37,724 women to the survey conducted by the European Union Agency for Fundamental Rights on violence against women [26]. Respondents to this survey were ever-partnered women, aged from 18 to 74 years old, from the 28 EU countries. The responses were collected following a two-stage clustered stratified sampling design with equal probability of selection of households within clusters; structured interviews were conducted in person [40]. The average response rate to the survey was 42.1%, ranging from 18.5% in Luxembourg to 84.0% in Hungary [40]. Quality control checks were made for 10% of the interviewed women [40]. A license for secondary data analysis was granted by the FRA for all the analyses (Reference No. 102577).

The sample used in this study comprised the responses from respondents who answered all of the items addressing psychological partner violence. Socio-demographical information of the sample by country can be found in Table 1.

**Table 1:** Sociodemographic characteristics of the sample in each country (*N* = 37,724)

	Age (%)				Income (%)				Self-perceived health		Experiences of child abuse (%)
	18–24	25–34	35–59	60+	Q1	Q2	Q3	Q4	M	SD	
Austria	12.4	21.3	45.1	21.1	34.1	23.0	26.6	16.3	1.77	0.86	26.7
Belgium	8.0	17.0	53.6	21.4	10.7	24.5	37.7	27.1	2.04	0.87	26.0
Bulgaria	4.9	13.5	44.2	37.3	12.5	39.1	35.0	13.5	2.34	1.00	27.7
Croatia	5.4	15.1	52.2	27.3	27.0	22.7	25.8	24.5	2.23	1.11	31.8
Cyprus	18.4	27.5	45.1	9.1	6.2	27.4	49.5	16.9	1.60	0.80	12.2
Czech Republic	9.8	16.6	49.0	24.6	29.8	29.2	22.8	18.2	2.06	0.89	32.3
Denmark	12.2	15.3	49.0	23.5	25.5	35.1	24.9	14.5	1.82	0.90	41.9
Estonia	5.9	12.7	47.0	34.4	28.0	23.6	26.1	22.3	2.56	0.92	48.4
Finland	5.9	15.5	45.2	33.5	19.3	22.4	27.6	30.7	2.07	0.79	52.2
France	5.4	14.7	57.0	22.9	52.7	32.5	9.7	5.1	1.99	0.84	44.9
Germany	5.7	13.3	55.9	25.2	23.5	26.1	29.2	21.3	2.14	0.84	42.8
Greece	7.4	16.4	50.9	25.2	21.9	40.7	33.1	4.3	1.69	0.88	23.7
Hungary	8.3	15.3	46.7	29.6	20.1	29.4	23.7	26.8	2.49	0.99	23.8
Ireland	6.2	20.1	49.7	24.1	39.7	30.3	20.9	9.2	1.69	0.83	27.7
Italy	4.4	14.6	60.8	20.1	62.5	30.0	5.4	2.0	2.23	0.76	33.6
Latvia	7.4	15.7	46.9	30.0	23.6	26.0	25.0	25.5	2.68	0.83	31.5
Lithuania	5.6	11.8	54.3	28.3	44.2	16.7	16.5	22.6	2.57	0.85	18.4
Luxembourg	3.8	14.0	63.7	18.5	15.5	32.6	31.4	20.5	1.94	0.89	44.0
Malta	3.9	13.6	52.3	30.1	30.8	41.3	20.6	7.2	2.06	0.66	23.4
Netherlands	3.2	12.1	59.5	25.2	18.9	28.7	26.9	25.6	2.15	0.77	33.0
Poland	9.9	23.7	47.5	18.8	10.4	24.7	30.7	34.2	2.23	0.98	17.6
Portugal	4.3	11.3	47.8	36.6	22.4	40.6	20.6	16.3	2.66	0.92	28.4
Romania	9.2	18.7	47.9	24.3	29.2	20.7	19.8	30.3	2.38	1.03	22.7
Slovakia	6.8	18.6	53.9	20.8	18.3	23.7	28.1	29.9	2.10	0.96	32.9
Slovenia	5.5	13.4	52.3	28.9	12.9	30.9	35.2	21.1	2.13	0.90	13.2
Spain	3.9	15.3	54.7	26.1	35.4	29.4	22.3	13.0	2.13	0.89	29.2
Sweden	2.7	10.8	54.3	32.2	3.5	27.2	23.6	45.7	1.93	0.92	43.2
United Kingdom	6.0	15.9	50.0	28.1	38.3	8.9	8.8	44.0	1.92	0.95	38.0

The percentages and descriptive statistics are unweighted. *M* Mean, *SD* Standard Deviation. Income: Q1 = under lowest quartile, Q2 = between lowest quartile and median, Q3 = between median and highest quartile, Q4 = above highest quartile

### Measures

#### Psychological violence

The FRA survey includes two sets of items addressing psychological IPVAW in the first part of the interview. The first set contains eight items assessing controlling behavior (e.g., “Insisting on knowing where she is in a way that goes beyond general concern”), and economic violence (e.g., “Preventing you from making decisions about family finances or from shopping independently”). The second set is comprised of five items evaluating emotional abuse (e.g, “Belittling or humiliating you in front of other people”). The response format for all the items was a 4-point Likert-type scale indicating the frequency of such behaviors (1: “Never”, 2: “Sometimes”, 3: “Often”, 4: “All the times”). Given that in most of the countries the frequencies of the upper two categories were extremely low (less than 2%, even when merged together), we decided to dichotomize the responses in order to set the same metric in all the items (0: “Never”, 1: “Sometimes or more often”).

### Validity evidence based on relations to other variables

We used the following variables to test the validity of the psychological IPVAW measure [41]:

- *Physical violence*. Physical IPVAW is assessed in the FRA survey with a set of eight items describing episodes of physical violence perpetrated by either the current or any previous partner (e.g., “Has your current/previous partner ever slapped you?”). The response format of the items was dichotomized (0: “Never”, 1: “Once or more times”). The factor scores of this measure were used for the validity analyses.
- *Sexual violence*. Sexual IPVAW is evaluated in the FRA survey with a set of four items addressing sexual violence committed by the current or any previous partner (e.g., “Has your current/previous partner made you take part in any form of sexual activity when you did not want to or you were unable to refuse?”). The responses to these items were also dichotomized (0: “Never”, 1: “Once or more times”). The factor scores of this measure were also used for the validity analyses.
- *Self-perceived health*. The FRA survey used a single item inquiring about the general health of the respondents at the beginning of the interview, using a 5-point Likert type graded response (from 1 = “Very Bad” to 5 = “Very Good”).
- *Experiences of child abuse*. This variable was assessed in the FRA survey using a set of 11 questions asking about experiences of childhood physical and sexual abuse before the age 15 (e.g., “Did an adult who was 18 years or over hit you very hard so that it hurt?”, “has an adult who was 18 years or over expose their genitals to you?”). If any of these questions were answered affirmatively, we considered that the participant had experienced abuse during their childhood.
- *Income*. The FRA survey includes a single item of reported income in each country (i.e., “under lowest quartile”, “between lowest quartile and median”, “between median and highest quartile”, “above highest quartile”). To answer this item, respondents informed the interviewer about their monthly income or chose between four income bands. These bands varied depending on the country to make the quartiles comparable across all EU member states [40] (e.g., in Austria: “up to €1,600”, “€1601–€2300”, “€2300–€3000”, “over €3000”).

### Statistical analyses

The main objective of this study is to assess the measurement invariance of the items addressing psychological IPVAW included in the FRA Survey, and to examine how the levels of this type of violence are distributed across the EU. To do so, we carried out the following analyses.

We first conducted a descriptive analysis of the set of items addressing psychological IPVAW, obtaining the mean, standard deviation, skew, and kurtosis statistics, as well as the correlation of each item with the rest of the scale (i.e., item-test corrected correlation). We then analyzed the latent structure of this measure carrying out a confirmatory factor analysis (CFA) for each country separately. We compared two models: a one-factor model, where all the items loaded onto a single factor, and a two-factor correlated models, in which all the items of the first set—controlling behavior and economic violence—loaded on one factor, and the items of the second set—emotional abuse—loaded onto a second factor. Although the first set of questions has items of controlling behavior and economic violence, we decided to maintain the structure used in the FRA survey. To this end, both types of items were included in the same factor, since there were not enough indicators to estimate a separate factor of economic violence—the FRA survey only included two items of economic violence, and a minimum of three indicators are usually required [42, 43]. Given the categorical nature of the data, we used weighted least squares means and variances adjusted (WLSMV) as the estimation method. The fit of the models was evaluated by a combination of fit indices: the comparative fit index (CFI), the Tucker-Lewis index (TLI), and the root mean squared error of approximation (RMSEA). CFI and TLI values above .95 are indicative of good fit [44], whereas RMSEA values below .08 and .06 were considered as mediocre and excellent fit, respectively [45]. The internal consistency of the resulting factors was assessed using Revelle’s omega total [46], as it does not assume tau-equivalence for the items [47]. Omega values above .70 are indicative of good internal consistency.

Once the latent structure of the scale was established, we carried out a series of multi-group confirmatory factor analyses (MG-CFA) to test the measurement invariance of the psychological IPVAW measure across the 28 EU countries. To this end, a series of nested models were evaluated: configural, metric and scalar invariance models [28]. In the configural invariance model, the same factor structure is applied for all countries, assuming no equality constraints for any parameters. The metric invariance model holds the item loadings to the same value across countries. The scalar invariance model constraints both item loadings and thresholds to be invariant across countries, ensuring that respondents from different countries with the same pattern of responses will obtain the same factor score. If the scalar invariance model is supported, then the factor means on the psychological IPVAW measure can be compared across countries.

To assess the fit of the invariance models, we computed the change in the CFI (ΔCFI) and RMSEA (ΔRMSEA), using the general guidelines of Cheung and Rensvold [48] and Chen [49]. These guidelines, however, were developed for continuous data, and thus the interpretation of such indices should be made with caution when dealing with categorical data [50]. For this reason, we used the cut-off values proposed by Meade, Johnson, and Brady [51], as it is currently the most conservative approach for assessing the change in the fit indices: ΔCFI ≤ .002 and ΔRMSEA ≤ .007. The performance of these cut-offs with categorical data tend to be similar to maximum likelihood based procedures when the sample size is large and the items are not normally distributed [50].

After establishing an invariant factorial model, we used the invariant factor scores of the psychological IPVAW measure to test validity evidence based on relationships to other variables in all countries. Pearson correlations were obtained between the psychological IPVAW measure and the measures of physical and sexual IPVAW. In addition, we computed a one-way ANOVA, testing differences in psychological IPVAW by self-perceived health, experiences of child abuse, and income. The size effect of the variables was assessed with the partial eta-squared statistic, using values above .01, .06, and .14, as indicative of small, medium, and large size effects, respectively [52].

Finally, we compared the factor means of psychological IPVAW across countries through a latent means analysis. This procedure takes into account the different weights of the items (i.e., item loadings) to measure the construct and all the constraints of the invariance analyses, leading to a more appropriate and sophisticated comparison of psychological IPVAW levels across all EU countries. We evaluated the magnitude of these cross-national comparisons using Cohen’s *d* statistic. Values of this statistic above .20, .50, and .80 indicate small, medium and large size effects, respectively [53]. In addition to Cohen’s *d*, we also computed the Cohen’s *U*_*3*_ statistic, which reflects the percentage of cases of one country that is higher than the average of another [54, 55]. Cohen’s *U*_*3*_ between each pair of countries can be found on the Additional file 1.

All analyses were conducted with the library *psych* of the statistical package R [46, 56], with the exception of the CFA and the MG-CFA, which were computed using *Mplus 8.3* [57].

## Results

### Descriptive analysis

All the items presented means close to zero, as well as high skew and kurtosis statistics, showing that most of the respondents reported never having experienced the episodes described by the items (Table 2). The item-test corrected correlations were high in general, indicating a strong relationship between the items.

**Table 2:** Descriptive statistics of the psychological violence items

	M	SD	Skew	Kurtosis	r_item-test_
Controlling Behavior and Economic Violence					
Try to keep you from seeing your friends?	0.18	0.38	1.68	0.81	0.73
Try to restrict your contact with your family of birth or relatives?	0.11	0.31	2.48	4.16	0.65
Insist on knowing where you are in a way that goes beyond general concern?	0.23	0.42	1.32	−0.27	0.72
Get angry if you speak with another man/woman?	0.22	0.42	1.33	−0.24	0.70
Become suspicious that you are unfaithful?	0.21	0.41	1.39	−0.05	0.68
Prevent you from making decisions about family finances and from shopping independently?	0.11	0.31	2.49	4.20	0.58
Forbid you to work outside the home?	0.05	0.21	4.26	16.16	0.47
Forbid you to leave the house, takes away your car keys or locks you up?	0.05	0.21	4.29	16.41	0.52
Emotional Abuse					
Belittled or humiliated you in front of other people?	0.19	0.39	1.62	0.61	0.66
Belittled or humiliated you in private?	0.26	0.44	1.10	−0.79	0.69
Done things to scare or intimidate you on purpose, for example by yelling and smashing things?	0.19	0.39	1.55	0.41	0.69
Made you watch or look at pornographic material against your wishes?	0.02	0.15	6.41	39.15	0.32
Threatened to hurt or kill someone you care about?	0.04	0.19	4.9	21.98	0.40

*M* Mean, *SD* Standard Deviation, *r*_*item-test*_ corrected item-test correlation. Skew and kurtosis standard error were below .01

### Confirmatory factor analysis and internal consistency

Two models where tested in each country: a one-factor model assuming that all the items are grouped onto a single factor, and a two-factor model, distinguishing between the controlling behavior and the emotional abuse items.

The one-factor model yielded a very good fit in all countries, showing excellent CFI and TLI values in the 28 EU countries (Table 3). The RMSEA, however, indicated only excellent fit in Bulgaria, Estonia, Finland, Ireland, Luxembourg, Malta, Romania, and Spain, presenting a mediocre model fit in the rest of the countries, with the exception of Croatia, where the RMSEA for the one-factor model was poor. Regarding the two-factor model, all the fit indices were excellent, showing almost a perfect fit. Nevertheless, the correlations between the two factors of this second model were very strong, yielding values around .85 in most of the countries (ranging from .73 in Croatia to .93 in Ireland), which in turn may be indicating that both factors are measuring the same construct. Given the suitability of both factorial solutions, we decided to keep both factor structures for the measurement invariance analysis in order to then choose the one better supported by the data.

**Table 3:** Confirmatory factor analysis fit indices by country

	One-factor model			Two-factor model		
	CFI	TLI	RMSEA	CFI	TLI	RMSEA
Austria	0.98	0.98	0.066	0.99	0.99	0.039
Belgium	0.99	0.98	0.070	0.99	0.99	0.049
Bulgaria	0.99	0.99	0.052	0.99	0.99	0.035
Croatia	0.99	0.98	0.101	0.99	0.99	0.056
Cyprus	0.98	0.98	0.071	0.99	0.99	0.040
Czech Republic	0.98	0.97	0.073	0.98	0.98	0.054
Denmark	0.97	0.97	0.071	0.98	0.98	0.048
Estonia	0.99	0.99	0.055	0.99	0.99	0.032
Finland	0.99	0.98	0.059	0.99	0.99	0.039
France	0.99	0.98	0.061	0.99	0.99	0.038
Germany	0.98	0.98	0.066	0.99	0.98	0.045
Greece	0.99	0.98	0.060	0.99	0.99	0.036
Hungary	0.99	0.99	0.080	0.99	0.99	0.043
Ireland	0.99	0.99	0.059	0.99	0.99	0.041
Italy	0.98	0.98	0.069	0.99	0.98	0.045
Latvia	0.98	0.98	0.070	0.99	0.99	0.036
Lithuania	0.98	0.97	0.074	0.99	0.99	0.039
Luxembourg	0.99	0.99	0.055	0.99	0.99	0.037
Malta	0.99	0.98	0.056	0.99	0.99	0.037
Netherlands	0.98	0.97	0.076	0.98	0.98	0.053
Poland	0.99	0.99	0.066	0.99	0.99	0.040
Portugal	0.99	0.98	0.069	0.99	0.99	0.038
Romania	0.99	0.99	0.058	0.99	0.99	0.033
Slovakia	0.99	0.99	0.069	0.99	0.99	0.045
Slovenia	0.99	0.98	0.067	0.99	0.99	0.037
Spain	0.99	0.99	0.056	0.99	0.99	0.030
Sweden	0.97	0.97	0.075	0.98	0.98	0.051
United Kingdom	0.99	0.99	0.063	0.99	0.99	0.038

*CFI* Comparative Fit Index, *TLI* Tucker-Lewis Index, *RMSEA* Root Mean Squared Error of Approximation

The internal consistency was very good in both models. In particular, the omega total for the one-factor solution was ω = .90 in the complete sample (ranging from .88 in Denmark to .93 in Bulgaria, Croatia, and Ireland). For the two-factor solution the omega total was ω = .91 (ranging from .88 in Denmark to .95 in Croatia).

### Measurement invariance

The analysis of measurement invariance supported the configural, metric, and scalar invariance models for the psychological IPVAW measure across all EU countries for the one-factor solution (Table 4). When the factor loadings were constrained to have the same value in all the countries, the metric model fit did not differ substantially from the configural model (∆CFI = .000, ∆RMSEA = .006). Similarly, constraining the item thresholds as well as the item loadings to be equal across countries did not substantially reduce the fit of the model (∆CFI = .001, ∆RMSEA = .002), supporting the scalar invariance model.

**Table 4:** Measurement invariance fit indices

Model	χ^2^	*df*	CFI	TLI	RMSEA [95% CI]
One-factor model					
Configural	11,855.74	1820	0.987	0.985	0.064 [0.063–0.065]
Metric	12,131.20	2171	0.987	0.987	0.058 [0.057–0.059]
Scalar	13,947.57	2360	0.985	0.986	0.060 [0.059–0.061]
Two-factor model					
Configural	6046.60	1792	0.995	0.993	0.042 [0.041–0.043]
Metric	8884.20	2143	0.991	0.991	0.048 [0.047–0.049]
Scalar	10,739.80	2440	0.989	0.990	0.050 [0.049–0.051]

*χ2* Adjusted chi-squared test for model fit, *df* Degrees of freedom, *CFI* Comparative Fit Index, *TLI* Tucker-Lewis Index, *RMSEA* Root Mean Squared Error of Approximation

Regarding the two-factor solution, however, the data only supported the configural invariance model. Constraining the loadings to be equal in all the countries reduced the fit of the metric invariance model (∆CFI = .004, ∆RMSEA = .006), above the ∆CFI .002 cut-off suggested by Meade et al. [51]. We thus decided to keep the one-factor solution as the latent structure of the psychological IPVAW measure for the rest of the analyses.

**Fig. 1:**
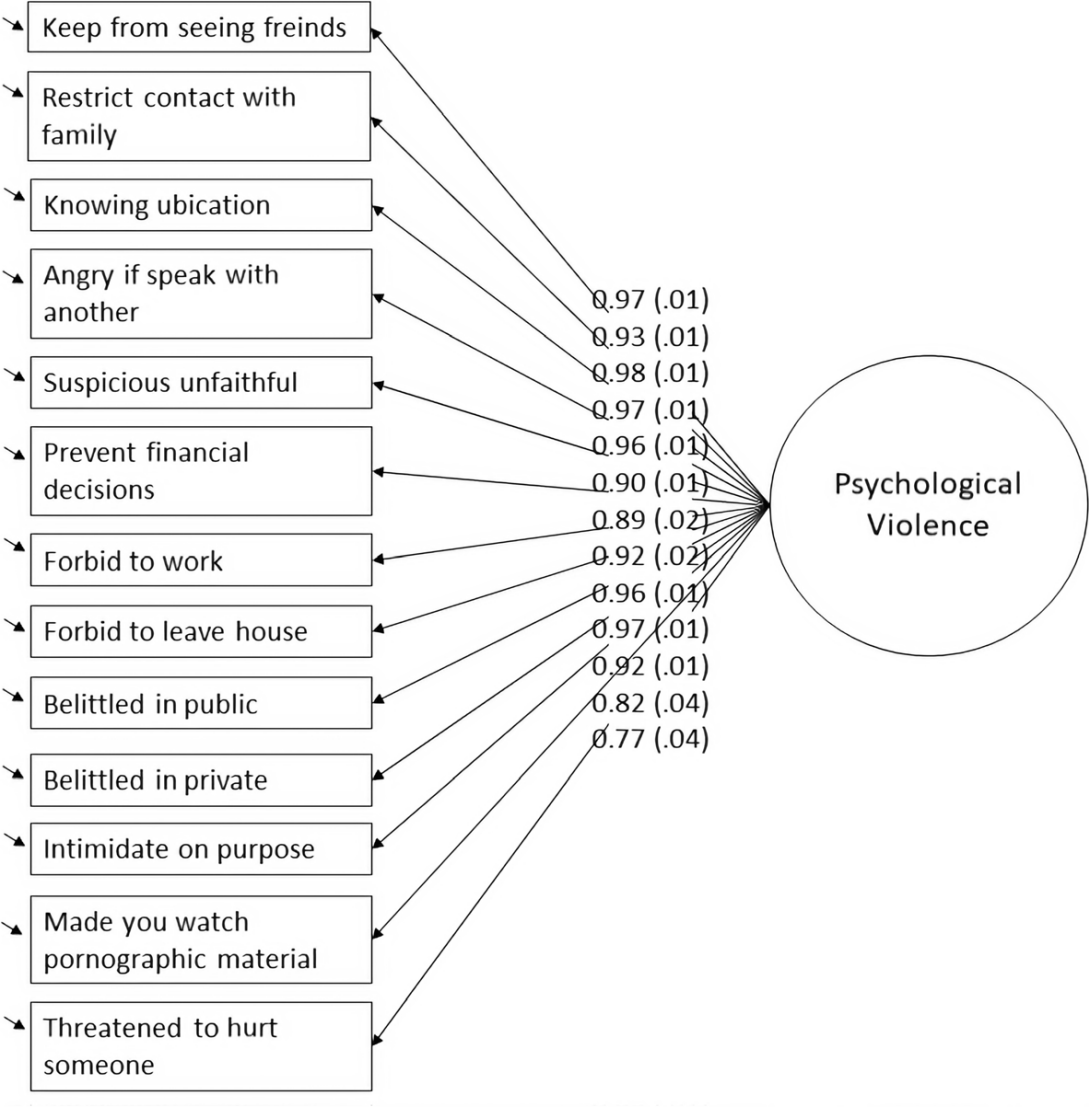
Multi-group confirmatory factor analysis: scalar invariance model. The standardized factor loadings belong to the reference country (i.e., Ireland). As in the standardized solution the factor variances are fixed to 1, there are slight differences in the decimals of the loadings in each country

The standardized item loadings of the one-factor model were high in general, with values above .90 for most of the items and low standard errors (Fig. 1). There were, however, small differences between the items, pointing out that not all the items contribute equally to the factor, and that some items were more relevant than others to assess the construct. Regarding the item thresholds, they were around 1 for most of the items, ranging from 0.60 for the item “Get angry if you speak with another man/woman?” to 2.11 for the item “Made you watch or look at pornographic material against your wishes?”, covering a wide area of the latent trait continuum. The items with the lowest factor loadings (“Made you watch or look at pornographic material against your wishes?”, and “Threatened to hurt or kill someone you care about?”), were also the items with the highest thresholds (i.e., 2.11 and 2.08, respectively), indicating that they were addressing more severe forms of violence but with somewhat less precision.

### Validity evidence based on relations to other variables

We used the factor scores of the invariant one-factor model for the validity analyses in all countries. We found a positive and strong relationship between the psychological IPVAW factor scores and the physical and sexual IPVAW factor scores (*r* = .85 and *r =* .75, respectively), indicating that those women with higher levels of psychological IPVAW also tend to show higher levels of physical and sexual IPVAW.

Significant differences with a small size effect were found in the psychological IPVAW scores when experiences of child abuse were taken into account (*F* (1) = 1548, *p* < .001, *η*^*2*^ = .040), as respondents with a background of child abuse showed higher psychological IPVAW levels. Significant differences were found in self-perceived health (*F* (4) = 47.4, *p* < .001, *η*^*2*^ = .001) and income (*F* (1) = 138.9, *p* < .001, *η*^*2*^ = .004), although the size effect of both variables was negligible.

### Latent means analysis

**Fig. 2:**
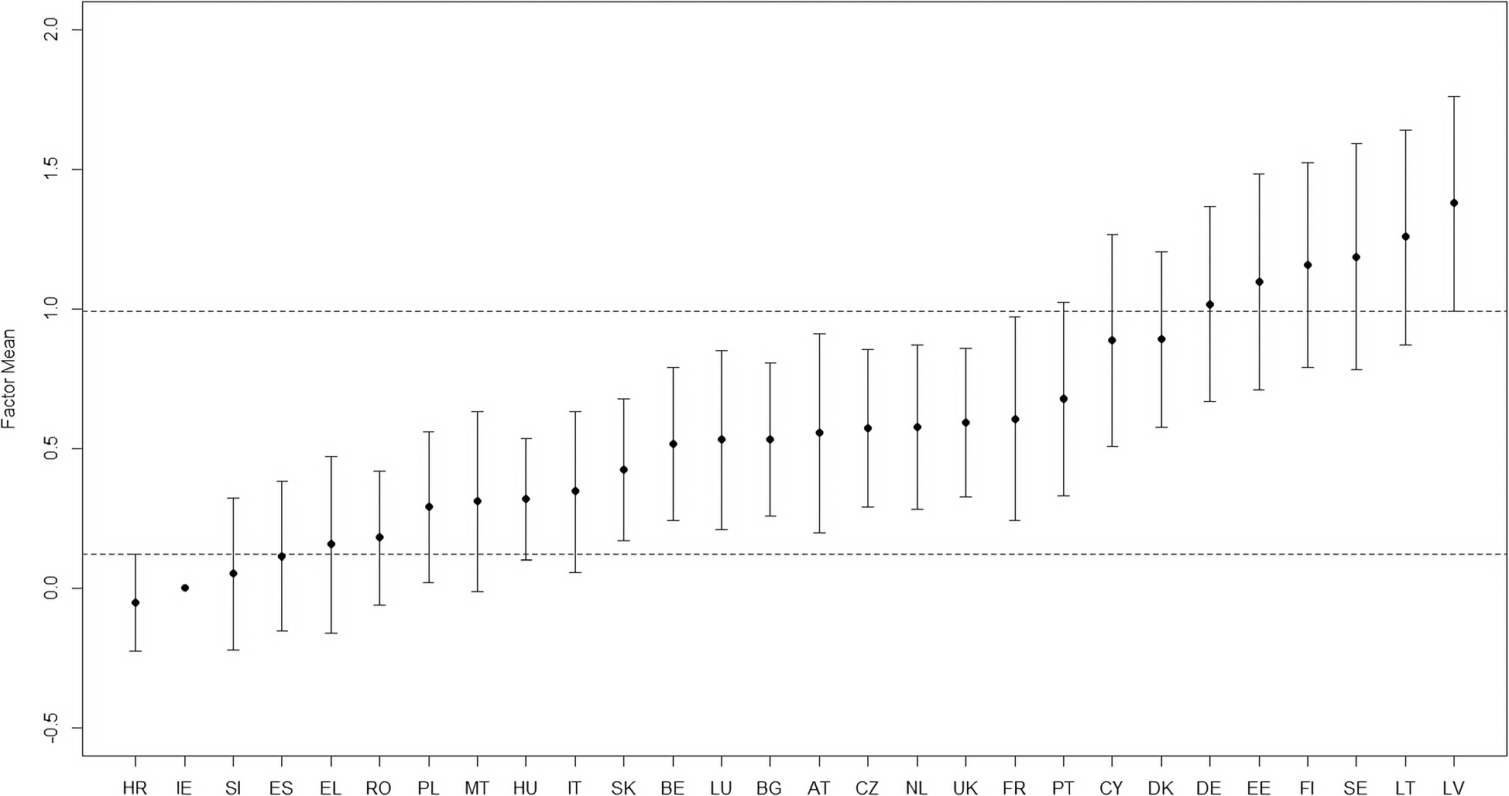
Psychological IPVAW latent means across the EU. IPVAW: Intimate partner violence against women. AT: Austria, BE: Belgium, BG: Bulgaria, CY: Cyprus, CZ: Czech Republic, DK: Denmark, EE: Estonia, FI: Finland, FR: France, DE: Germany, EL: Greece, ES: Spain, HR: Croatia, HU: Hungary, IE: Ireland, IT: Italy, LT: Lithuania, LV: Latvia, LU: Luxembourg, MT: Malta, NL: Netherlands, PL: Poland, PT: Portugal, RO: Romania, SE: Sweden, SI: Slovenia, SK: Slovakia, UK: United Kingdom. Ireland is the reference country

After determining that the psychological IPVAW measure is psychometrically sound and establishing an invariant model across all EU countries, the means of the psychological violence factor can now be properly compared across countries by carrying out a MG-CFA. We used Ireland as the reference group, as it was the country with the lowest rates of psychological IPVAW [26]. We fixed the latent mean for this country to zero and its variance to one, whereas in the rest of the countries these parameters were freely estimated. The model converged normally and showed a good fit (CFI = .986, TLI = .986, RMSEA = .061 [.060–.062]). The standardized latent mean of each country represents the difference in standard deviations from Ireland. The estimated latent means for each country and their confidence intervals are displayed in Fig. 2.

Croatia showed a lower latent mean than Ireland, although these differences were not significant (*z* = − 0.05, *p* = .554, *d* = 0.06). Similarly, no significant differences were found between Ireland and Slovenia (*z* = 0.05, *p* = .771, *d* = 0.05), Spain (*z* = 0.11, *p* = .405, *d* = 0.11), Greece (*z* = 0.16, *p* = .339, *d* = 0.14), and Romania (*z* = 0.18, *p* = .144, *d* = 0.17). We found significant differences with a large size effect between Ireland and Latvia (*z* = 1.38, *p* < .001, *d* = 0.93), Lithuania (*z* = 1.26, *p* < .001, *d* = 0.88), Finland (*z* = 1.16, *p* < .001, *d* = 0.81), and Sweden (*z* = 1.19, *p* < .001, *d* = 0.80).

The confidence intervals (CI) of the psychological IPVAW factor means were overlapped between many of the EU countries, showing that there were no significant differences between them. However, we grouped the countries using the CI of Croatia and Latvia, the countries with the lowest and highest levels of psychological IPVAW. Therefore, countries whose factor mean fell within the CI of Croatia showed on average lower levels of psychological IPVAW (Ireland, Slovenia, and Spain). On the other hand, countries with factor means that fell within the CI of Latvia presented higher levels of this type of violence (Lithuania, Finland, Sweden, and Estonia). The rest of the countries fell in a middle area, with countries like Greece or Romania closer to those countries with the lowest levels of psychological IPVAW, and others like Germany or Denmark closer to those countries with the highest levels of this type of violence.

The psychological IPVAW factor scores can also be compared between each pair of countries. As an illustrative example, we compare the factor scores of Sweden, Austria, and Spain—countries with high, moderate and low levels of psychological IPVAW— using Cohen *U*_*3*_ statistic. We found small differences between Sweden and Austria (*d* = .41, *U*_*3*_ = .659), showing that the 65.9% of the Swedish sample has higher values in the psychological IPVAW factor than the average of the Austrian sample. We found moderate differences between Sweden and Spain (*d* = .74, *U*_*3*_ = .770), with 77.0% of the Swedish sample presenting higher scores in this factor than the average of the Spanish sample. Smaller differences were found between Austria and Spain (*d* = .36, *U*_*3*_ = .641), as the 64.1% of the Austrian sample had higher factor scores in the psychological IPVAW factor than the mean of the Spanish sample. A table detailing the comparisons between each pair of countries and as its associated size effect is provided in the supplementary information section.

## Discussion

Although psychological IPVAW is one of the most extended forms of partner violence, little attention has been paid to the cross-national comparability of the data used in large population and health surveys. In this study we tackled this issue through a set of analyses to test the measurement invariance of the set of questions addressing this type of violence in the FRA survey, and then examining how psychological IPVAW levels were distributed across all EU countries.

The first set of analyses aimed to assess the psychometric properties (i.e., latent structure, reliability, and validity) of the set of questions assessing psychological IPVAW in each of the EU countries. The initial CFA suggested that the items of this measure could be mapping either a one- or a two-factor latent structure. Currently, there is not a clear consensus on how psychological IPVAW should be conceptualized and measured in cross-national research, and although a two-factor structure seems to be theoretically preferred, the number of studies addressing the latent structure of this type of IPVAW is rather scarce [2, 58]. Even though previous studies have established a two-factor structure for psychological IPVAW, distinguishing between emotional abuse and other forms of controlling behavior with similar items, the correlation between emotional abuse and controlling behavior is usually strong, which in turn may indicate than a one-factor structure could be sufficient to account for the variability of the construct [12, 23, 58]. This idea was supported by the measurement invariance analyses, where we found that metric and scalar invariance only held under the one-factor structure, underlining that the items addressing emotional abuse and controlling behavior of the FRA survey could be grouped into a single factor across the EU (i.e., psychological IPVAW). The resulting factor showed high internal consistency, with ω values above .85 in all countries.

Regarding the validity evidence based on relations with other variables, our results pointed out that, as expected, psychological IPVAW was strongly related to physical and sexual IPVAW. The co-occurrence of psychological IPVAW with other forms of violence is a well-known phenomenon in the literature, and it is often considered as an antecedent of physical IPVAW [11, 13]. In addition, we found that the women who reported experiences of child abuse also presented higher levels of psychological IPVAW, which is in line with previous research indicating that women who have been victimized in childhood have higher risk of being victimized as adults [35, 36, 59].

The second set of analyses aimed to test the measurement invariance of the psychological IPVAW measure used in the FRA survey across all EU countries. Establishing measurement invariance is a necessary prerequisite in cross-national research before conducting any comparison between countries [31]. To this end, we carried out a series of MG-CFA models to examine whether the psychological IPVAW measure is comparable across the 28 EU countries, testing configural, metric, and scalar invariance. We were able to hold these three invariance models (i.e., same factors with equal loadings and thresholds) under the one-factor latent structure across countries, despite the difficulties frequently found into achieving this with many groups [60, 61]. This may be due to the behavioral nature of the items used in the FRA survey, since they address the frequency of concrete episodes which may be difficult to misinterpret (e.g., “insisting on knowing where you are in a way that goes beyond general concern”, “doing things to scare or intimidate her on purpose”).

We were able to make appropriate and valid comparisons between countries once an invariant model was established for the psychological IPVAW measure. This is one of the main strengths of this study, since it allowed us to conduct a latent means analysis to compare the factor means of each country, a procedure that unlike computing the raw prevalence, takes into account the latent structure of the construct and the weight of each item to assess it [28, 29]. Our findings showed that there were almost no differences between most of the countries, as the confidence intervals of the factor means overlapped, indicating that the levels of psychological IPVAW were quite similar between them. We found, however, substantial differences between the countries with higher and lower levels of psychological IPVAW. For example, countries like Latvia, Lithuania, Finland, and Sweden presented average levels of psychological IPVAW significantly higher than countries such as Croatia, Ireland, Slovenia and Spain. Given the close relationship between different forms of IPVAW, this finding may further support the idea of the Nordic paradox [62, 63], as despite being the EU member states with the highest levels of gender equality, the Nordic countries —Denmark, Sweden, and Finland—are also among the EU countries with the highest levels of psychological IPVAW.

This study has some limitations. The first one is the cross-sectional nature of the survey design, as it did not allow us to evaluate measurement invariance across different periods of time, neither to monitor whether the levels of psychological IPVAW change or remain constant over time. A second limitation is the tradeoff associated with the dichotomization of the items of the psychological IPVAW measure. This transformation was necessary to set the same metric for all the items, but we lose a small percentage of information (i.e., less than a 2%) about the most continued forms of this type of violence. The third limitation of this study refers to the assessment of the latent structure of the psychological IPVAW measure used in the FRA survey. Although this measure includes three forms of psychological violence (i.e., controlling behavior, economic violence, and emotional abuse), it was not possible to test a three-factor structure differentiating between these three aspects due to the reduced number of items addressing economic violence included in the FRA survey, since estimating a third factor with only two indicators loading on it could yield an unstable and unreliable solution [42, 43]. Our results are restricted to the measure used in the FRA survey, and thus it should be taken with caution when generalizing to other psychological IPVAW measures. The latent structure of this construct is still a matter of debate, and further research is needed to test different models using more items mapping the different forms of psychological IPVAW [58]. The fourth limitation concern the estimation method used to establish the measurement invariance of the set of questions included in the FRA survey, that is, WLSMV for categorical indicators. We decided to rely on the CFI and RMSEA fit indices to assess the goodness of fit of the invariance models rather than using chi-squared based tests, which are known to be sensible to large sample sizes. However, utilizing fit indices to study the measurement invariance with categorical data could lead to higher rates of Type I errors, assuming an invariant model when actually the instrument is non-invariant [50]. To tackle this issue, instead of the usual cut-offs for the TLI and RMSEA proposed by Chen [49], we decided to use the cut-off values proposed by Meade et al. [51], a much more restrictive approach. A fifth limitation of this study is the wide range of the confidence intervals of the psychological IPVAW factor means, which suggest that this measure could be improved in order to yield more accurate estimations of the IPVAW levels. Self-selection bias is another limitation, since only a 42% of the participants agreed initially to be interviewed. This is also reflected in the wide variability in the response rates across the EU countries, with some countries presenting response rates lower than 30% (e.g., Luxembourg, Netherlands, or Sweden), whereas others showed response rates above 60% (e.g., Cyprus, Hungary, or Latvia) [40]. Finally, the results of this study are limited to IPVAW, and the question of whether measurement invariance holds across countries when the perpetrators of the psychological violence against women are non-partners remains unexplored.

## Conclusion

This study underlined the importance of using appropriate and robust statistical methods to test the measurement invariance of the measures used in large population surveys. Although psychological IPVAW is one the most frequent form of intimate partner violence, and one of the main risk factors of physical IPVAW, to the best of our knowledge, no previous study has addressed the measurement invariance of the measures used in any large survey to assess this construct. Our findings showed that the psychological IPVAW measure used in the FRA survey is invariant across all EU countries, allowing us to conduct a more refined analysis of how the levels of this variable are distributed across the EU. This is an important step towards a rigorous assessment of cross-national differences in psychological IPVAW, and further research is needed to evaluate the role that the country plays in accounting for the differences across EU countries [64].

## Supplementary information

**Additional file 1.** Psychological IPVAW comparisons between countries.

## Data Availability

The data that support the findings of this study are available from the UK Data Service (https://www.ukdataservice.ac.uk), but restrictions apply to the availability of these data, which were used under license for the current study, and so are not publicly available. The dataset details are the following: Title: European Union Agency for Fundamental Rights: Violence Against Women Survey, 2012: Special Licence Access. Alternative title: FRA VAW Survey, Persistent identifier: 10.5255/UKDA-SN-7730-1.

## Abbreviations

CFA: Confirmatory factor analysis
CFI: Comparative fit index
CI: Confidence interval
CTS: Conflict Tactics Scale
EU: European Union
FRA: European Union Fundamental Rights Agency
IPVAW: Intimate partner violence against women
MG-CFA: Multi-group confirmatory factor analysis
RMSE: Root mean squared error of approximation
TLI: Tucker-Lewis index
WLSMV: weighted least squares means and variances adjusted
